# Rational development of synergistic combinations of chemotherapy and molecular targeted agents for colorectal cancer treatment

**DOI:** 10.1186/s12885-018-4712-z

**Published:** 2018-08-13

**Authors:** Diego Tosi, Esther Pérez-Gracia, Salima Atis, Nadia Vié, Eve Combès, Mélissa Gabanou, Christel Larbouret, Marta Jarlier, Caroline Mollevi, Adeline Torro, Maguy Del Rio, Pierre Martineau, Céline Gongora

**Affiliations:** 1 Institut régional du Cancer de Montpellier (ICM), 208 avenue des Apothicaires, 34298 Montpellier, France; 2 Institut de Recherche en Cancérologie de Montpellier (IRCM, Inserm U1194), 34298 Montpellier, France

**Keywords:** Colorectal cancer, Drug combinations, Phosphokinome, Irinotecan, Synergistic effect

## Abstract

**Background:**

The irinotecan-induced phosphokinome changes in colorectal cancer (CRC) cells were used to guide the selection of targeted agents to be tested in combination with irinotecan.

**Methods:**

Phosphokinome profiling with peptide arrays of tumour samples from nude mice xenografted with HT29 cells and treated or not with an effective dose of irinotecan was used to identify signalling pathways activated by irinotecan treatment. Then, drugs targeting these pathways were combined in vitro with irinotecan to test potential synergistic effect. The interactions between these drug combinations were assessed by a dose matrix approach. Confirmation of the most potential combination has been confirmed in vivo in xenografted mice.

**Results:**

Irinotecan induced in vivo the activation of AKT and MEK1 phosphorylation. The dose matrix approach showed that BKM120 (PI3K inhibitor) and MEK162 (MEK inhibitor) are synergistic in vitro and in vivo with a cytostatic and cytotoxic effect, while combination of BKM120 and irinotecan or MEK162 and irinotecan are only additive or even antagonistic. However, the triple combination of SN38, BKM120 and MEK162 showed a better synergistic effect that BKM120 and MEK162, indicating that the cells need to inhibit both AKT and ERK pathways to become more sensitive to irinotecan-based chemotherapies.

**Conclusion:**

Analysis of chemotherapy-induced phosphokinome changes helps to elucidate the mechanisms of drug resistance and to guide the selection of targets for combination therapies with synergistic activity.

**Electronic supplementary material:**

The online version of this article (10.1186/s12885-018-4712-z) contains supplementary material, which is available to authorized users.

## Background

In recent years, personalized medicine in which the specific genetic makeup of each patient is taken into account to target specific pathways has achieved significant therapeutic successes in oncogene-addicted cancers, such as HER2-overexpressing breast cancer, non-small cell lung cancer with EGFR mutations or melanoma with BRAF mutations [1–4]. However, this strategy has been partly disappointing in the large majority of malignancies because of the extremely complex crosstalk between cellular pathways and the enormous adaptive capabilities of tumour cells [5, 6].

For long time, new targeted drugs have been developed as single agents or as add-on treatments to a standard therapy (particularly chemotherapy). However, recently, combinations of targeted agents have started to be considered as the best hope for effective therapies with durable effects [7]. Yet, scarce data are available about the mechanistic interactions of combinations of targeted agents and chemotherapy. In addition, the interaction effects (in terms of synergism, antagonism or additivity) between targeted drugs and cytotoxic agents are not frequently investigated, as well as the drug doses that maximize the efficacy of combinations. In this setting, preclinical studies could facilitate the selection of drugs, doses and schedules for early clinical trials.

The PI3K/AKT/mTOR and MAPK pathways are among the most important intracellular signalling cascades in cancer cells [8, 9]. Their activation depends mainly on the stimulation of membrane receptor tyrosine kinases (RTKs) by mitogenic factors that act in a paracrine or autocrine manner. Due to the frequent concomitant activation of the PI3K/AKT/mTOR and MAPK pathways and the multiple interaction between these cascades [6, 10, 11], their combined inhibition has been tested in preclinical studies, often with interesting results [12–14]. Clinical trials are analysing combinations of pharmacological inhibitors of these two pathways [15–17]. Preliminary results show signs of activity, but associated with significant toxicities [18].

Consistent evidence demonstrated that increased expression and activation of membrane RTKs upstream of the PI3K/AKT/mTOR and MAPK pathways are a common compensatory response to pharmacological inhibition of intracellular kinases [19–21]. On the other hand, the chemotherapy-induced modifications in the activity of these pathways have been less frequently investigated [22]. We previously showed that in the colorectal cancer (CRC) cell line HCT116, SN38 (the active metabolite of irinotecan) induces the activation of AKT and MEK, with an increase of their phosphorylation by 2- and 7-fold, respectively [23]. This suggests that phosphokinome alterations could be a mechanism used by CRC cells to escape irinotecan cytotoxic effect. We thus hypothesized that AKT and MEK activation is used by CRC cells to promote their survival upon treatment, and that a synergistic effect could be obtained in CRC cells by associating irinotecan with agents that inhibit AKT and MEK, thus allowing to significantly reduce the drug doses.

## Methods

### Cell lines and drugs

The HCT116 (CCL-247), SW48 (CCL-231), SW480 (CCL-228), LS174T (CCL-188) and HT29 (HTB-38) colon adenocarcinoma cell lines from the American Type Culture Collection (ATCC, Manassas, Virginia) were grown in RPMI-1640 medium supplemented with 10% foetal calf serum (FCS) and 2 mmol/L L-glutamine at 37 °C in a humidified atmosphere with 5% CO_2_. The SN38–resistant HCT116 clones were obtained as previously described [24]. Briefly, the reference SN38–sensitive HCT116 clone (HCT116-s) was incubated with 10 nmol/L or 15 nmol/L SN38 and cloned to obtain the HCT116-SN6 clone and the HCT116-SN50 clone, respectively. All cell lines were cultured in drug-free medium at least for five days before any experiment.

### 3D cell culture system

Ultra-low attachment, round-bottomed 96-well plates (Corning Costar) were used for spheroid formation. HCT116 cells were seeded at a density of 150 cells/well. Cells aggregated and merged to form 3D spheroids within 24-72 h. Spheroids were then grown in the presence of BKM120 (75, 150, 300, 600 and 1200 nM) and MEK162 (0.3, 1, 3, 10, 30, 90, 270 nM) (added at day 4 and day 10 of culture) and experiments ended 14 days after seeding. Images of wells were taken with a phase-contrast microscope using a 10X objective. Cell viability was assessed with the CellTiter-Glo Luminescent Cell Viability Assay (Promega, Madison, WI, USA). After addition of 100 μl of CellTiter Glo reagent to each well for 10 min, luminescence was measured on a 1450 MicroBeta TriLux Luminescence microplate reader (Perkin Elmer).

### In vivo testing

All in vivo experiments were performed under the supervision of an accredited researcher (Dr. Céline Gongora, accreditation #34–142) in compliance with the French regulations and ethical guidelines for experimental animal studies. Female athymic *nu/nu* mice were purchased from Harlan Laboratories and used at 6 to 8 weeks of age and were maintained in a specific and opportunistic pathogen-free (SOPF) facility in an accredited establishment (Agreement No. C34–172-27). All animals were healthy and drug naïve before experiments, and their weight was 21 to 30 g. To evaluate antitumor effect of drugs, 1 × 10^6^ HT29 or HCT116 tumour cells were injected subcutaneously (s.c.) in the left flank of each mouse. Tumours were detected by palpation and measured periodically with callipers. When tumours reached 100 mm^3^, mice were randomized in groups of 6 to 9 animals, to be treated with the indicated drugs or with vehicle (0.9% sodium chloride, controls).

The irinotecan stock solution was diluted in 0.9% sodium chloride and administered by intraperitoneal (i.p.) injection (four injections of 20 mg/kg, one every 4 days). Mice in the control group received 0.2 mL of 0.9% sodium chloride solution (i.p. injection) according to the same schedule.

BKM120 and MEK162 (0.3, 1, 3, 30 and 0.3, 1, 3, 10, 30 mg/kg, respectively) were administered by i.p. injection to tumour-bearing mice 5 times per week. Mice in the control group received 0.2 mL of 0.9% sodium chloride solution according to the same schedule.

Mice were euthanized by cervical dislocation when the tumour volume reached 1500 mm^3^ and explanted tumour xenografts were frozen for phosphokinome analysis.

### Phosphokinome profiling

Xenografts from nude mice were isolated and proteins extracted as follows. Tumours were cut and lysed in 500 μL of M-PER lysing buffer complemented with protease and phosphatase inhibitors and then homogenized with beads using the MixerMill apparatus. Extracts were centrifuged and proteins in the supernatants were quantified with the Bradford assay. Kinase phosphorylation was then profiled using the PamGene technology. Specifically, phosphotyrosine kinase (PTK) and phosphoserine/threonine kinase (STK) activity were assessed using the PTK and STK PamChip arrays and 1 and 0.5 μg of protein extracts, respectively, on a PamStation12.

On each array, 144 peptides with tyrosine or serine/threonine phosphorylation sites were immobilized. FITC-labelled anti-phosphotyrosine or anti-phosphoserine/threonine antibodies were used to detect peptide phosphorylation. Peptide phosphorylation was monitored by taking images with a charge coupled device (CCD) camera in combination with the Evolve software v1.2 (PamGene) that allows the real-time recording of the reaction kinetics. After array washing, fluorescence was detected at different exposure times (20, 50, 100 and 200 ms). Luminescence signal acquisition was performed over 200 ms and the 100 ms values estimated by linear regression were normalized to the mean of the single array means and used for analysis. A Z score for the treated versus control sample values was calculated.

### Evaluation of protein expression by western blotting

After counting, cells were lysed in SDS buffer (bromophenol blue, 5% β-mercaptoethanol, 2% SDS, 10% glycerol, 62.5 mM Tris-HCl pH 6.8). Extracts were treated with benzonase, boiled for 5 min and separated by SDS-PAGE. Proteins were electro-transferred to nitrocellulose membranes (Amersham Pharmacia Biotech, Uppsala, AB, Sweden). Primary antibodies were against AKT, ERK and MEK1 and their phosphorylated forms, and against GAPDH and tubulin (loading controls). Secondary antibodies were horseradish peroxidase (HRP)-conjugated anti-mouse or anti-rabbit IgGs (Sigma Aldrich). Proteins were detected by enhanced chemoluminescence (ECL) with the ECL detection system from GE Healthcare Life Sciences (Buckinghamshire, UK) and images recorded using the G/BOX iChemi imaging system (Syngene).

### Drug sensitivity assay

Cell growth inhibition and cell viability after incubation with SN38, BKM120 and MEK162 were assessed using the sulforhodamine B (SRB) assay. Exponentially growing cells (1000 cells/well) were seeded in 96-well plates in RPMI-1640 medium supplemented with 10% FCS. After 24 h, serial dilutions of the tested drugs were added and each concentration was tested in duplicate or triplicate. After 96 h, cells were fixed with 10% trichloroacetic acid and stained with 0.4% SRB in 1% acetic acid (Sigma Aldrich). SRB fixed to the cells was dissolved in 10 mmol/L Tris–HCl and absorbance at 540 nm was read using an MRX plate reader (Dynex, Inc., Vienna, VA, USA).

### Quantification of the interaction effect

The interaction between the drugs tested in vitro and in vivo was investigated with a dose matrix test, in which increasing doses of each single drug were tested with all possible dose combinations of the other drugs. For each dose combination, the percentage of surviving cells expected in the case of additivity was calculated according to the Loewe equation [25]:

$$ \frac{d_A}{IC_{x,A}}+\frac{d_B}{IC_{x,B}}=1 $$

where *d*_*A*_ and *d*_*B*_ are the doses of the inhibitory drugs A and B, and *IC*_*X,A*_ and *IC*_*x,B*_ their isoeffective doses that result in an effect of *x*%. The percentage of expected surviving cells in the case of effect independence was calculated according to the Bliss equation [25]:

$$ {fu}_c={fu}_A{fu}_B $$

where *fu*_c_ is the expected fraction of cells unaffected by the drug combination in the case of effect independence, and *fu*_A_ and *fu*_B_ are the fractions of cells unaffected by treatment *A* and *B,* respectively. The generalized form of the Bliss equation for a combination of *n* treatments:

$$ {fu}_c=\prod \limits_{i=1}^n{fu}_i $$

was used to analyse the interaction effect of the combination of three drugs. The difference between the *fu*_c_ value and the fraction of living cells in the cytotoxicity test was considered as an estimation of the interaction effect, with positive values indicating synergism and negative values antagonism. In addition to this point-by-point estimation, a synthetic index of interaction for the entire dose matrix (called combination index, CI) was calculated using the method proposed by Lehár [26–28]:

$$ CI=\ln {f}_A\ \ln {f}_B\sum \left({Z}_O-{Z}_E\right) $$

where *f*_*A*_ and *f*_*B*_ are the dilution factors used in the cytotoxicity assay for drugs *A* and *B,* respectively, and *Z*_*O*_ and *Z*_*E*_ are the matrices of the survival percentage for the experimental data and for the corresponding Loewe additivity or Bliss independence data, respectively. CI is a positive-gated, effect-weighted volume over Loewe additivity or Bliss independence, adjusted for the variable dilution factors *f*_*A*_ and *f*_*B*_. R scripts used for interaction analysis are available in Additional file 1.

### Cell-death analysis

Trysined cells were fixed with 4% paraformaldehyde and permeabilised with methanol. Then the cells were incubated with anti-caspase-3 antibody (CST-9661) followed with a secondary anti-rabbit-Alexa Fluor488 from Invitrogen (A1108). The analysis were performed on FC500 Beckman coulter.

### Proliferation assay

Cell proliferation was measured by incorporating 5-ethynyl-2′-deoxyuridine (EdU) into DNA during active DNA synthesis (i.e., the last 16 h of culture). After cell fixation and permeabilization in 75% ethanol/PBS, incorporated EdU was labelled and detected with the Click-iT® EdU Alexa Fluor® 488 Flow Cytometry Assay Kit (Invitrogen, Carlsbad, CA, USA). Analyses were done on a FC500 Beckman Coulter Flow Cytometer. EdU-positive cells were quantified using the Flow Jo analysis software (Treestar Inc., Ashland, Oregon, USA).

### Cell cycle analysis

Cells were seeded in 25 cm^2^ flasks (2 × 10^4^ cells/flask). After 24 h, cells were incubated with 1 μmol/L BKM120, 0.1 μmol/L MEK162 or both for 96 h. One million cells were then pelleted, washed with PBS, fixed in 75% ethanol and stained with 40 μg/mL of propidium iodide solution containing 100 μg/mL of RNase). Cell cycle distribution was determined with a FC500 Beckman Coulter Flow Cytometer using the FL-3 channel. Cells were gated on a dot plot that displayed DNA pulse-peak versus DNA-pulse area to exclude doublets. Cell cycle distributions were illustrated using the Flow Jo analysis software (Treestar Inc).

### Statistical analysis

For in vitro experiments, data were compared using the unpaired Student’s t test.

For the mouse models, a linear mixed-regression model, containing both fixed and random effects, was used to determine the relationship between tumour growth and number of days after grafting. Ata were first transformed using the natural log scale to better fit the assumptions of the linear mixed model. The fixed part of the model included variables corresponding to the number of post-graft days and the different treatments. Interaction terms were built into the model; random intercepts and random slopes were included to take time into account. The coefficients of the model were estimated by maximum likelihood and considered significant at the 0.05 level. The log-rank test was used to compare survival curves between groups. Statistical analysis was performed using the STATA 10.0 software (StataCorp).

## Results

### Phosphokinome remodeling after irinotecan treatment

**Fig. 1:**
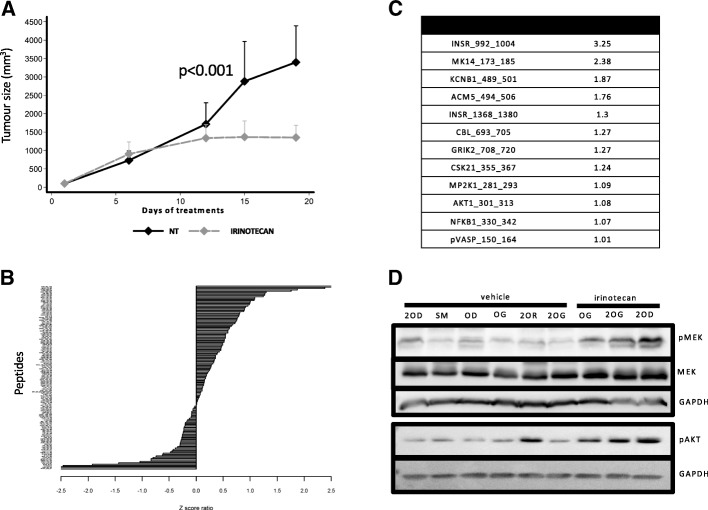
Effect of irinotecan treatment on the phosphokinome profile of HCT116 cell xenografts. (**a**) Tumour growth in nude mice treated with 20 mg/kg irinotecan or vehicle (NT) (*n* = 6/group). Each growth curve represents the mean of tumor growth volume for mice of the corresponding group. (**b**) Z-scores of the luminescence values of individual target peptides from PTK and STK PamChip arrays using irinotecan-treated and control samples. (**c**) List of PTK and STK peptides with a Z score > 1 and the corresponding Z-score value; peptide names are provided according to the UniProt nomenclature. (**d**) Western blot assay showing the increase of MEK and AKT phosphorylation in tumour samples from xenografted mice treated with irinotecan or vehicle (saline)

To evaluate the effect of irinotecan treatment on CRC phosphokinome profile, tumours from nude mice xenografted with HT29 cells and treated with irinotecan (*n* = 6) or saline solution (n = 6) for 19 days (Fig. 1a) were analysed using PTK and STK PamChip arrays. Analysis of the phosphorylation changes of the tested kinase (described using Z-scores) (Fig. 1b) indicated that INSR, AKT and MEK1 (MP2K1) were among the kinases with a Z-score greater than 1 (Fig. 1c). The highest Z-score was attributed to insulin receptor (INSR), that could be partially responsible for MEK1 and AKT activation. The irinotecan-induced AKT and MEK1 phosphorylation increase was confirmed by western blotting of HT29 cell xenografts (Fig. 1d).

### In vitro evaluation of the synergism of two-drug combinations

**Fig. 2:**
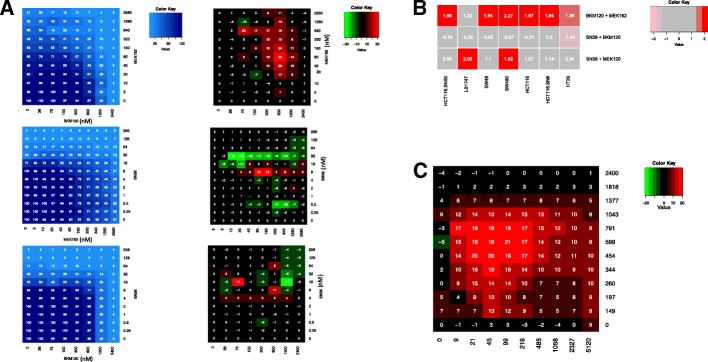
In vitro synergism analysis for two-drug combinations of BKM120, MEK162 and SN38. (**a**) Experimental survival data (SRB assay; left) and synergism (right) matrices for HCT116 cells incubated with the indicated two-drug combinations at the indicated doses. In the blue matrices, values indicate the percentage of surviving cells. In the synergism matrices, the green to red scale is used to indicate antagonist combinations (green) and synergistic combinations (red). The black cells show additive combinations. (**b**) Combination indices for all the CRC cell lines incubated with two-drug combinations of BKM120, MEK162 or SN38. (**c**) Synergism matrix for the BKM120-MEK162 combinations focused on the synergism area. Table 1 shows the features of each cell line

We decided to target AKT and MEK in order to inhibit the effect of INSR activation and, at the same time, to evaluate drug combinations suitable for clinical application. Then, the interaction of BKM120 (PI3K inhibitor), MEK162 (MEK inhibitor) and SN38 was evaluated using a full-range dose matrix approach and SRB cytotoxicity tests for two-drug combinations (BKM120-MEK162, MEK162-SN38, and BKM120-SN38) in seven molecularly characterized CRC cell lines (Table 1). First, the combinations, using seven different BKM120 doses, ten MEK162 doses and eleven SN38 doses, were analysed in HCT116 cells (Fig. 2a) and then in the other cell lines (summary in Fig. 2b). Results are presented as the percentage of cell survival (blue matrix) and values of the additive, synergistic and antagonistic effects (black, red and green matrices, respectively) (Fig. 2a for HCT116 cells). A synergistic interaction was observed mainly with the BKM120-MEK162 combinations (Fig. 2a, upper panels), with a mean combination index of 1.94 (range: 1.22 to 2.27) in all the tested cell lines (Fig. 2b). Drug combinations including chemotherapy showed less positive interactions. A slight synergistic interaction was detected between SN38 and MEK162 (Fig. 2a, center matrices), with a mean CI of 1.10 (range: 0.04 to 2.05; Fig. 2b). Combinations of SN38 and BKM120 were mainly additive (Fig. 2a, bottom panels), with a mean CI of - 0.31 (range: - 1.43 to 0.60; Fig. 2b). The distribution of the interaction effects was not homogeneous over the dose matrices. For instance, analysis of the matrices for the BKM120-MEK162 combinations (Fig. 2a, upper panels) showed focal areas with higher synergy that were located in dose regions corresponding to 90% to 30% of cell survival with BKM120 and 80% to 65% with MEK162. Analysis of the matrices for the BKM-SN38 combinations showed focal areas of antagonism located in dose regions that corresponded to 85% to 70% of cell survival with BKM120 and 95% to 70% with SN38 (Fig. 2a, bottom panels). No clear correlation was detected between the drug interactions and the KRAS and p53 mutational status or sensitivity to irinotecan of the used CRC cell lines (Table 1 and Fig. 2b). Further analysis of the BKM120-MEK162 combination with more doses (Fig. 2c) showed that the more synergistic doses were between 21 and 219 μM for MEK162 and 344 and 791 μM for BKM120.

**Table 1:** Molecular characteristics and drug resistance of cell lines

Cell lines	KRAS	BRAF	p53	PI3K	Drug resistance
HCT116	G13D	wt	wt	H1047R	EGFRi
HCT116-SN6	G13D	wt	wt	H1047R	SN38/EGFRi
HCT116-SN50	G13D	wt	wt	H1047R	SN38/EGFRi
SW480	G12 V	wt	R273H/P309S	wt	EGFRi
LS174T	G12D	wt	wt	H1047R	EGFRi
HT29	wt	V600E	R273H	wt	EGFRi
SW48	wt	wt	wt	wt	none

*Abbreviations*: *wt* wild type, *EGFRi* EGFR inhibitors

**Fig. 3:**
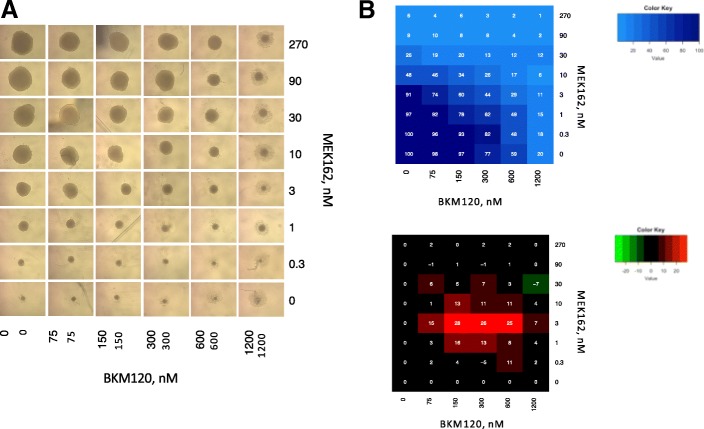
In vitro testing of the BKM120-MEK162 synergism in HCT116 cell spheroids. (**a**) Microphotographs of spheroids after 14 days of incubation with BKM120 and MEK162 at the indicated doses. (**b**) Experimental survival data (top) and synergism (bottom) matrices

Then, the same approach was used to assess the BKM120-MEK162 interaction in HCT116 spheroids (Fig. 3a). These experiments confirmed the presence of an area of significant synergism (i.e., more than 15% of cytotoxicity due to the synergistic interaction) in dose regions that corresponded to 98% to 59% of cell survival with BKM120 and 97% to 91% with MEK162, with a peak of synergism (i.e., ≥25% of cytotoxicity due to the synergistic interaction) in the dose regions of 97% to 59% of cell survival for BKM120 and 91% for MEK162 (Fig. 3b). The cytotoxic effect detected in the dose area of intense synergism corresponded to 60% to 29% of cell survival, and the synergistic interaction accounted for 28% to 25% of the drug effect, which corresponded to 70% and 35% of the total cytotoxic effect, respectively. The most synergistic doses were between 150 and 600 nM for BKM120 and 3 nM for MEK162.

### Functional analysis of the BKM120-MEK162 combination at synergistic doses

**Fig. 4:**
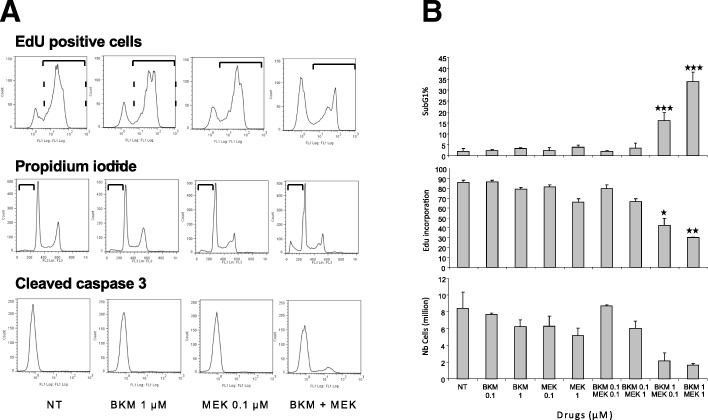
Functional analysis of the effect of BKM120 and/or MEK162 at the indicate doses in HCT116 cells. (**a**) FACS profiles for EdU incorporation, propidium iodide staining (late apoptosis marker) and presence of cleaved caspase 3 (early apoptosis marker) and (**b**) the corresponding mean intensity values in the various experimental conditions. NT, not treated

As the BKM120-MEK162 combination was the most efficient in SRB assays, its functional effects were evaluated in HCT116 cells (Fig. 4). Apoptosis analysis by quantifying the percentage of propidium iodide-positive cells (i.e., cells in the subG1 phase of the cell cycle, representing late apoptosis) and of cleaved caspase 3-positive cells (early apoptosis) (Fig. 4a and b) showed that the BKM120 and MEK162 combination at synergistic doses (1 μM and 0.1 μM, respectively) increased apoptosis, compared with each drug on its own or absence of treatment (NT). Similarly, analysis of cell proliferation by EdU incorporation showed that proliferation was inhibited only in cultures incubated with the drug combination and not with each drug alone (Fig. 4a and b). In agreement, cell number was reduced only by the MEK162-BKM120 treatment (Fig. 4b). Overall these results indicate that the BKM120-MEK162 combination has synergistic cytotoxic and cytostatic effects.

### In vivo evaluation of BKM120-MEK162 synergism

**Fig. 5:**
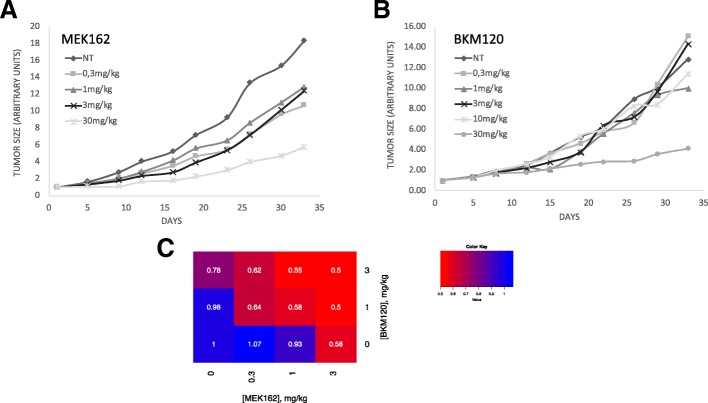
In vivo evaluation of the interaction of the BKM120-MEK162 combination. (**a** and **b**) Growth curves of HCT116 xenografts in nude mice treated with vehicle (NT) or different doses of MEK162 (**a**), or of BKM120 (**b**). Each growth curve represents the mean of tumor growth volume for mice of the corresponding group. Experimental groups were treated and assessed in the following order: first the NT group with vehicle, and then the other groups according to the concentrations of BKM120 in increasing order and, for the groups treated with the same concentration of BKM120, according to the concentrations of MEK162 in increasing order. (**c**) Dose matrix representing the fractional tumour growth inhibition in mice treated with different dose combinations of BKM120 and MEK162

To evaluate in vivo the effect of the BKM120-MEK162 combination, preliminary experiments were carried out to select doses associated with a limited effect of each single drug on tumour growth in xenografted mice (Fig. 5a and b). To this aim, groups of six mice were treated or not (saline, NT) with different concentrations of MEK162 (0.3, 1, 3 and 30 mg/kg) or BKM120 (0.3, 1, 3, 10 and 30 mg/kg). Treatments did not induce any side effect. For MEK162, the best antitumor effect (compared with control) was observed with the 30 mg/kg dose, and a mild tumour growth inhibition with 0.3, 1 and 3 mg/kg. On the other hand, 30 mg/kg was the only BKM120 dose that induced tumour growth inhibition. Then, mice xenografted with HCT116 cells (*n* = 6 per group) were treated with the BKM120-MEK162 combination (12 possible dose combinations, BKM120 alone (0, 0.3 or 3 mg/kg) or MEK162 alone (0, 0.3, 1 or 3 mg/kg i.p.) (Fig. 5c). A synergistic effect was detected with BKM120-MEK162 combinations at doses that did not show any significant effect as single drugs (i.e., a fold increase ratio between tested dose and control higher than 5%). Particularly, combining 0.3 mg/kg BKM120 with 0.3 or 1 mg/kg MEK162 led to a reduction in the median tumour volume fold increase of 24% and 21%, which is nearly entirely due to the synergistic interaction between these drugs (Fig. 5c).

### Evaluation of the SN38, BKM120-MEK162 combination synergism

**Fig. 6:**
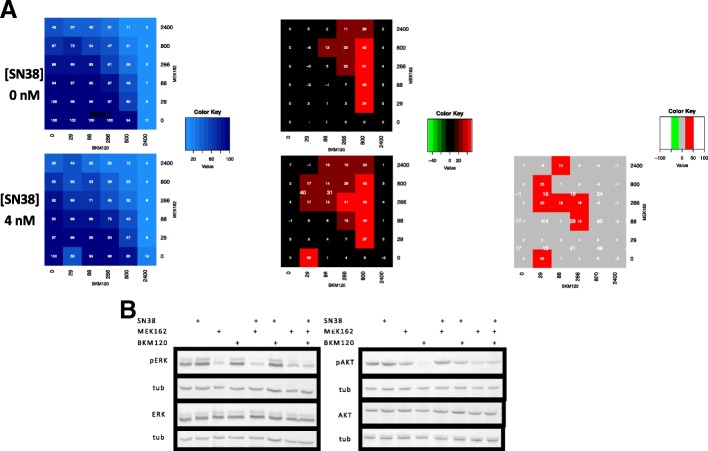
In vitro synergism analysis of the three-drug combination (BKM120, MEK162 and irinotecan) in cells. (**a**) On the left, the blue matrices show the observed values of cytotoxicity (SRB assay) for BKM120 and MEK162 without (top) or with 4 nM SN38 (bottom). Middle panels, synergy matrices show the percentage of cytotoxicity due to drug synergism. Right panel, the matrix shows the percentage of cytotoxicity due to irinotecan addition. (**b**) Western blot analysis showing the inhibition of ERK and AKT phosphorylation in HCT116 cells incubated with BKM120, MEK162 and SN38 (alone or in combination as indicated)

Finally, as irinotecan induces AKT and MEK phosphorylation in CRC cells (Fig. 1c), the interaction of the SN38, BKM120-MEK162 combination was evaluated in HCT116 cells using the SRB cytotoxicity assay and a three-dimensional dose matrix with increasing doses of the three drugs (five doses for BKM120, five doses for MEK162 and one dose for SN38) (Fig. 6a). Again, an important synergistic effect (red squares on the synergy matrices) were observed at doses not associated with significant single-drug activity (detailed in the blue survival matrix). Importantly, this effect was due to the three-drug combination because addition of 4 nM SN38 (inactive dose on its own) to BKM120 and MEK162 increased the cytotoxicity over the dose areas where synergism was detected for the other two drugs, and expanded the synergism areas towards lower doses of BKM120 and MEK162. Western blot analysis showed that in HCT116 cells incubated with the three-drug combination (BKM120, MEK162 and SN38), AKT and MEK phosphorylation were reduced (Fig. 6b).

## Discussion

Historically, the rationale for combining chemotherapy and targeted agents in medical oncology is frequently based on the add-on model, in which a targeted agent with antitumor activity is added to a backbone chemotherapy regimen that constitutes the standard treatment for that cancer. Preclinical screening is frequently performed with the aim of elucidating the interaction effects between chemotherapy agents and targeted drugs in order to select at least additive combinations, but frequently the mechanisms of action of the drugs are considered as independent.

**Fig. 7:**
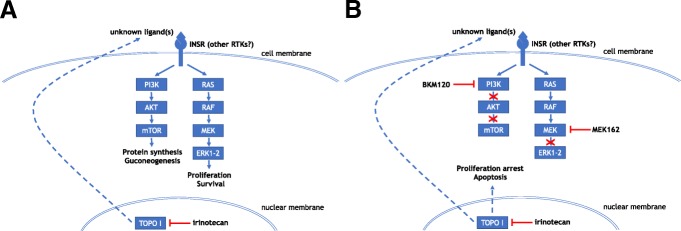
Schematic representation of the putative synergy mechanism between irinotecan, BKM 120 and MEK162. (**a**) Irinotecan treatment induce an increase of phosphorylation of INSR, which in turn activate PI3K/AKT/mTOR and RAS/RAF/MEK/ERK pathways and stimulate tumor cell survival and growth. (**b**) The combination of irinotecan, BKM120 and MEK162 abrogates the effect of IR activation and favors tumor cell proliferation arrest and apoptosis

In this study, we demonstrate that in CRC cells, irinotecan strongly activates the PI3K/AKT/mTOR and MAPK pathways, at least in part via the IR, and that the combination of irinotecan with both a PI3K inhibitor and a MEK inhibitor induces a synergistic effect in terms of cytotoxic activity (Fig. 7). This shows that activation of mitogen-dependent cancer cell signalling could constitute a response to the cancer cell system perturbation caused by chemotherapy, and could be involved in rescuing cancer cells. In addition, we demonstrate that the synergistic effect of the three-drug combination is observed with irinotecan doses associated with low activity when given as single drug, and with PI3K and MEK inhibitors at doses that lack consistent single-agent activity. In this case, synergism has the obvious advantage of allowing reducing the doses of the drugs used in combination, thus potentially decreasing also their toxicity.

Synergistic interactions between targeted drugs based on the induced dependence of cancer cells to a pathway activated by phosphokinome rewiring (and thus not associated with new genetic alterations) have been described in preclinical models of solid and haematological cancers. For instance, it was shown that in PTEN-deficient prostate cancer cell lines, inhibition of the PI3K pathway increases androgen receptor signalling, while androgen receptor inhibition activates AKT signalling [29]. In CRC cells with mutated BRAF, vemurafenib (inhibitor of mutated BRAF) induces an upregulation of EGFR that neutralizes vemurafenib effect. Conversely, the vemurafenib and cetuximab (EGFR inhibitor) combination is highly effective [19, 20]. These and our results clearly show that phosphokinome rewiring could, in some case, render ineffective the pharmacological inhibition of an oncogenic driver. Therefore, extensive profiling of functional adaptive responses might provide a rational base for combined targeted treatments, as described in our and in previous studies. For instance, a quantitative proteomic approach to assess kinome activity changes in response to MEK inhibition in triple-negative breast cancer (TNBC) cells and in genetically engineered mouse models (GEMMs) [21] allowed showing that MEK inhibition causes acute ERK activity loss, resulting in rapid c-MYC degradation, which in turn induces the expression and activation of several RTKs. RTK stimulation can overcome MEK2 inhibition, but not MEK1 inhibition, thus reactivating ERK and producing drug resistance. The inhibitor-induced RTK profile led to testing a combination treatment that produced GEMM tumour apoptosis and regression, while single agents were ineffective. These synergistic interactions could be considered as a form of synthetic lethality that is dependent on phosphokinome rewiring.

The complex mechanisms underlying synergistic interactions due to this type of induced synthetic lethality make the choice of doses to be tested difficult, because synergism can often be detected only when lower (and thus less or not at all effective) drug doses are used, as observed in our experimental set-up. Similarly, in the study cited above ([21] most of the kinases activated during MEK inhibitor-induced phosphokinome remodelling could be targeted by sorafenib, a BRAF inhibitor. The combination of sorafenib and the MEK inhibitor showed a clear synergistic interaction, because similar reduction of cell proliferation was obtained using a 10-fold lower dose of MEK inhibitor when combined with a dose of sorafenib that is not effective in its own. Moreover, we show that the effect of two single-agent drugs can be less than additive at certain dose combinations. This highlights the need of a characterization of the mechanistic interactions between chemotherapy and targeted agents to avoid hazardous choices in selecting drug combinations for clinical development. In addition, during functional phosphokinome remodelling in response to a perturbing stimulus, the sequence of drug administration could influence the phosphokinome changes and the efficacy of the drug combination. Convincing data in TNBC cell lines showed that EGFR inhibition dramatically sensitizes a subset of these cell lines to DNA damage if the drugs are given sequentially, but not simultaneously [22]. Transcriptional, proteomic and computational analyses of signalling networks in drug-treated cells revealed that the enhanced efficacy results from dynamic network rewiring of an oncogenic signature that is maintained by active EGFR signalling. These data highlight the need to understand the dynamics of network connectivity and to individuate changes in phosphokinome that could be targeted by combinations treatments.

The results of the present study have implications for the preclinical screening of drug combinations that include chemotherapy drugs and targeted agents. Indeed, profiling phosphokinome changes after treatment by irinotecan was the necessary step to identify appropriate targets for combination treatment. Our results also have multiple implications for the design of early clinical trials to test drug combinations. In this setting, selecting the dose combinations with synergistic interactions is challenging, because combination toxicity and single-agent efficacy cannot always be a useful guide and dose escalation may not be the more appropriate design. In addition, pharmacokinetics could influence such interactions, because drug concentration variations can significantly modify the phosphokinome status and then the interaction effect of combinations over time between drug administrations. Therefore, the drug dosing schedule should take into account the pharmacodynamic aspects of the interaction, and this because, although chemotherapy is expected to act by inducing a genotoxic hit (concentration-dependent effect) and the target drugs should introduce a signalling-perturbation hit (time-dependent effect), indeed chemotherapy could result in both.

## Conclusions

The availability of an ever-increasing number of targeted agents and of actionable targets requires rational strategies for developing combination treatments. Analysis of phosphokinome changes induced by chemotherapy could help to elucidate the mechanisms of escape, and orient towards potential targets for combination regimens with synergistic activity, and doses and schedules of combination regimens containing targeted agents.

## Additional file

Additional file 1: R script for interaction effect determination for two-drug combinations according to the Loewe equation. (DOCX 30 kb)

## Abbreviations

CCD: Charge coupled device
CI: Combination index
CRC: Colorectal cancer
ECL: Enhanced chemoluminescence
EdU: 5-ethynyl-2′-deoxyuridine
EGFRi: EGFR inhibitors
GEMM: Genetically engineered mouse model
HRP: Horseradish peroxidase
NT: Not treated
PTK: Phosphotyrosine kinase
RTKs: Receptor tyrosine kinases
SRB: Sulforhodamine B
STK: Phosphoserine/threonine kinase
TNBC: Triple-negative breast cancer
wt: Wild type
