# Supplementary material for: Rational development of synergistic combinations of chemotherapy and molecular targeted agents for colorectal cancer treatment
Source: BMC Cancer. 2018 Aug 13;18:812. doi: 10.1186/s12885-018-4712-z (PMC6090616; doi:10.1186/s12885-018-4712-z)
Supplement: Supplementary file 1 — R script for interaction effect determination for two-drug combinations according to the Loewe equation. (DOCX 30 kb) [file 12885_2018_4712_MOESM1_ESM.docx]

**Additional file 1 R script for interaction effect determination for two-drug combinations according to the Loewe equation.**

## data

setwd("/Users/User/My data")

name <- "figure name" # figure file name

name_a <- "a" # x-axis drug

name_b <- "b" # y-axis drug

data <- read.csv("data.csv", header=FALSE)

data

m <- as.matrix(data)

data_matrix <- round(m[1:(nrow(m) - 1), c(2:ncol(m))], 0)

data_matrix

dose_a <- m[nrow(m), c(2:ncol(m))]

dose_a

dose_b <- rev(m[,1])[-1]

dose_b

dimnames(data_matrix) <- (list(rev(dose_b), dose_a))

data_matrix

## cytotoxic curve parameters estimation for single drugs

# functions

R1 <- function(D, mi, d, p){

# Hill equation

# R = ma * (D / d)^-p / (1 + (D / d)^-p) - mi

# = ma * (d / D)^p / (1 + (d / D)^p) - mi

# ma is the maximum value of D, i.e. when x = 0

# mi is the minimum value of D, i.e. when x = ∞

# d is the value of D when R = (ma - mi) / 2

# p is a slope parameter

((100 - mi) * d^p) / (d^p + D^p) + mi

}

R1inv <- function(R, mi, d, p){

# Inverse of R1 function

# valid if R > mi

if (R <= mi)

return(Inf)

else

return(d * ((100 - R) / (R - mi))^(1 / p))

}

estimates_R10 <- function(X, Y){

# Start estimates for fitting

mi <- min(Y)

p <- 2

tmp <- abs(((100 - mi) / 2) - (Y - mi))

d <- X[tmp == min(tmp)][1]

return(list(mi, d, p))

}

estimates_R1 <- function(X, Y, lower = 0.1){

# Estimates for fitting

E0 <- estimates_R10(X, Y)

mi0 <- E0[[1]]

d0 <- E0[[2]]

p0 <- E0[[3]]

modele <- nls(Y ~ R1(X, mi, d, p), start = list(mi = mi0, d = d0, p = p0), trace = TRUE, control = nls.control(maxiter = 1000))

mi <- summary(modele)$coef[1, 1]

d <- summary(modele)$coef[2, 1]

p <- summary(modele)$coef[3, 1]

if(mi <= lower) return(estimates_R2(X, Y, lower))

if(mi > 0) return(list(mi, d, p))

}

estimates_R2 <- function(X, Y, lower = 0.1){

# Estimates for fitting

E0 <- estimates_R10(X, Y)

mi0 <- 0

d0 <- E0[[2]]

p0 <- E0[[3]]

modele <- nls(Y ~ R1(X, 0, d, p), start = list(d = d0, p = p0), trace = TRUE, control = nls.control(maxiter = 1000))

d <- summary(modele)$coef[1, 1]

p <- summary(modele)$coef[2, 1]

return(list(0, d, p))

}

plotR1 <- function(X, Y){

E_R1 <- estimates_R1(X, Y)

curve(R1(x, E_R1[[1]], E_R1[[2]], E_R1[[3]]), from = 1, to = max(X), col = 4, xlab = "Dose", ylab = "Surviving fraction (%)", log = "x", xlim = c(1, max(X)), ylim = c(0, 100), las = 1)

par(new = T)

plot(X, Y, col = 4, xlab = " ", ylab = " ", log = "x", xlim = c(1, max(X)), ylim = c(0, 100), las = 1)

curve(R1(x, E_R1[[1]], E_R1[[2]], E_R1[[3]]), from = 0, to = max(X), col = 4, xlab = "Dose", ylab = "Surviving fraction (%)", xlim = c(0, max(X)), ylim = c(0, 100), las = 1)

par(new = T)

plot(X, Y, col = 4, xlab = " ", ylab = " ", xlim = c(0, max(X)), ylim = c(0, 100), las = 1)

}

# estimations for drug a

inhibition_a <- data_matrix[nrow(data_matrix), ]

inhibition_a

estimates_a <- estimates_R1(dose_a, inhibition_a)

estimates_a

par(mfrow = c(2, 2))

plotR1(dose_a, inhibition_a)

# estimations for drug b

inhibition_b <- rev(data_matrix[, 1])

inhibition_b

estimates_b <- estimates_R1(dose_b, inhibition_b)

estimates_b

plotR1(dose_b, inhibition_b)

## Loewe additive effect estimation

f <- function(E, doseA, doseB){

# Loewe additivity equation

# 1 = (da / Dxa) + (db / Dxb) = (da / f^-1(fa(x))) + (db/f^-1(fb(x)))

# x is equieffective dose

# doseA is actual dose of drug A

# doseB is actual dose of drug B

mina <- estimates_a[[1]]

da <- estimates_a[[2]]

pa <- estimates_a[[3]]

minb <- estimates_b[[1]]

db <- estimates_b[[2]]

pb <- estimates_b[[3]]

doseA / R1inv(E, mina, da, pa) + doseB / R1inv(E, minb, db, pb) - 1

}

dx <- data.frame(A = rep(dose_a, length(dose_b)), B = as.vector(matrix(rep(rev(dose_b), length(dose_a)), byrow = TRUE, nrow = length(dose_a))))

dx <- as.matrix(dx)

Loewe_values_NA <- apply(dx, 1, function(r)

{

tryCatch(uniroot(function(x) f(x, r["A"], r["B"]), c(0.00001, 99.99999))$root, error = function(e) return(NA))

}

)

Loewe_values_NA

Loewe_matrix_NA <- matrix(Loewe_values_NA, nrow = length(dose_b), ncol = length(dose_a), byrow = TRUE, dimnames = list(rev(dose_b), dose_a))

Loewe_matrix_NA["0", "0"] <- 100

Loewe_matrix_NA

Loewe_values <- apply(dx, 1, function(r)

{

tryCatch(uniroot(function(x) f(x, r["A"], r["B"]), c(0.00001, 99.99999))$root, error = function(e) return(100))

}

)

Loewe_values

Loewe_matrix <- matrix(Loewe_values, nrow = length(dose_b), ncol = length(dose_a), byrow = TRUE, dimnames = list(rev(dose_b), dose_a))

Loewe_matrix["0", "0"] <- 100

Loewe_matrix

difference_matrix <- Loewe_matrix - data_matrix

dimnames(difference_matrix) <- list(rev(dose_b), dose_a)

difference_matrix

## calculation of combination index according to Lehar's method

# dilution factor of drug a

dfa <- dose_a[length(dose_a)] / dose_a[length(dose_a) - 1]

dfa

# dilution factor of drug b

dfb <- dose_b[length(dose_b)] / dose_b[length(dose_b) - 1]

dfb

CI <- (log(dfa) * log(dfb)* sum(difference_matrix, na.rm = TRUE)) / 100

CI

## graphics

library(gplots)

pdf(paste(name, ".pdf", sep = ""))

# experimental value matrix

f1 <- heatmap.2(data_matrix, dendrogram ="none", col=colorRampPalette(c("dodgerblue1", "navy"))(10), Rowv = NA, Colv = NA, cellnote = data_matrix, key = TRUE, density.info = "none", trace = "none", notecol = "white", labRow = rev(dose_b), labCol = c(dose_a), xlab = name_a, ylab = name_b, cexRow = 1.2, cexCol = 1.2)

# expected value matrices

f2a <- heatmap.2(Loewe_matrix_NA, dendrogram = "none", col=colorRampPalette(c("dodgerblue1", "navy"))(10), Rowv = NA, Colv = NA, cellnote=round(Loewe_matrix), key= TRUE, density.info = "none", trace = "none", notecol = "white", labRow = rev(dose_b), labCol = c(dose_a), xlab = name_a, ylab = name_b, cexRow = 1.2, cexCol = 1.2)

f2b<- heatmap.2(Loewe_matrix, dendrogram = "none",col = colorRampPalette(c("dodgerblue1", "navy"))(10), Rowv = NA, Colv = NA, cellnote = round(Loewe_matrix), key = TRUE, density.info = "none", trace = "none", notecol = "white", labRow = rev(dose_b), labCol = c(dose_a), xlab = name_a, ylab = name_b, cexRow = 1.2, cexCol = 1.2)

# interaction matrices

f3 <- heatmap.2(difference_matrix, dendrogram = "none", col = greenred(10), Rowv = NA, Colv = NA, cellnote = round(difference_matrix), key = TRUE, density.info = "none", trace = "none", notecol = "white", labRow = rev(dose_b), labCol = c(dose_a), xlab = name_a, ylab = name_b, cexRow = 1.2, cexCol = 1.2, symbreaks = T)

f4 <- heatmap.2(difference_matrix, dendrogram = "none", col = greenred, Rowv = NA, Colv = NA, cellnote = round(difference_matrix), key = TRUE, density.info = "none", trace = "none", notecol = "white", labRow = rev(dose_b), labCol = c(dose_a), xlab = name_a, ylab = name_b, cexRow = 1.2, cexCol = 1.2, symbreaks = T, breaks = c(seq(-25, 25, 5)))

dev.off()

**Supplementary Material 2. R script for interaction effect determination for three-drug combinations according to the Bliss equation.**

## data

setwd("/Users/User/My data")

name <- "figure name" # figure file name

name_a <- "a" # x-axis drug

name_b <- "b" # y-axis drug

data <- read.csv("data.csv", header=FALSE)

data

m <- as.matrix(data)

data_matrix <- round(m[1:(nrow(m) - 1), c(2:ncol(m))], 0)

data_matrix

dose_a <- m[nrow(m), c(2:ncol(m))]

dose_a

dose_b <- rev(m[,1])[-1]

dose_b

dimnames(data_matrix) <- (list(rev(dose_b), dose_a))

data_matrix

## cytotoxic curve parameters estimation for single drugs

# functions

R1 <- function(D, mi, d, p){

# Hill equation

# R = ma * (D / d)^-p / (1 + (D / d)^-p) - mi

# = ma * (d / D)^p / (1 + (d / D)^p) - mi

# ma is the maximum value of D, i.e. when x = 0

# mi is the minimum value of D, i.e. when x = ∞

# d is the value of D when R = (ma - mi) / 2

# p is a slope parameter

((100 - mi) * d^p) / (d^p + D^p) + mi

}

R1inv <- function(R, mi, d, p){

# Inverse of R1 function

# valid if R > mi

if (R <= mi)

return(Inf)

else

return(d * ((100 - R) / (R - mi))^(1 / p))

}

estimates_R10 <- function(X, Y){

# Start estimates for fitting

mi <- min(Y)

p <- 2

tmp <- abs(((100 - mi) / 2) - (Y - mi))

d <- X[tmp == min(tmp)][1]

return(list(mi, d, p))

}

estimates_R1 <- function(X, Y, lower = 0.1){

# Estimates for fitting

E0 <- estimates_R10(X, Y)

mi0 <- E0[[1]]

d0 <- E0[[2]]

p0 <- E0[[3]]

modele <- nls(Y ~ R1(X, mi, d, p), start = list(mi = mi0, d = d0, p = p0), trace = TRUE, control = nls.control(maxiter = 1000))

mi <- summary(modele)$coef[1, 1]

d <- summary(modele)$coef[2, 1]

p <- summary(modele)$coef[3, 1]

if(mi <= lower) return(estimates_R2(X, Y, lower))

if(mi > 0) return(list(mi, d, p))

}

estimates_R2 <- function(X, Y, lower = 0.1){

# Estimates for fitting

E0 <- estimates_R10(X, Y)

mi0 <- 0

d0 <- E0[[2]]

p0 <- E0[[3]]

modele <- nls(Y ~ R1(X, 0, d, p), start = list(d = d0, p = p0), trace = TRUE, control = nls.control(maxiter = 1000))

d <- summary(modele)$coef[1, 1]

p <- summary(modele)$coef[2, 1]

return(list(0, d, p))

}

plotR1 <- function(X, Y){

E_R1 <- estimates_R1(X, Y)

curve(R1(x, E_R1[[1]], E_R1[[2]], E_R1[[3]]), from = 1, to = max(X), col = 4, xlab = "Dose", ylab = "Surviving fraction (%)", log = "x", xlim = c(1, max(X)), ylim = c(0, 100), las = 1)

par(new = T)

plot(X, Y, col = 4, xlab = " ", ylab = " ", log = "x", xlim = c(1, max(X)), ylim = c(0, 100), las = 1)

curve(R1(x, E_R1[[1]], E_R1[[2]], E_R1[[3]]), from = 0, to = max(X), col = 4, xlab = "Dose", ylab = "Surviving fraction (%)", xlim = c(0, max(X)), ylim = c(0, 100), las = 1)

par(new = T)

plot(X, Y, col = 4, xlab = " ", ylab = " ", xlim = c(0, max(X)), ylim = c(0, 100), las = 1)

}

# estimations for drug a

inhibition_a <- data_matrix[nrow(data_matrix), ]

inhibition_a

estimates_a <- estimates_R1(dose_a, inhibition_a)

estimates_a

par(mfrow = c(2, 2))

plotR1(dose_a, inhibition_a)

# estimations for drug b

inhibition_b <- rev(data_matrix[, 1])

inhibition_b

estimates_b <- estimates_R1(dose_b, inhibition_b)

estimates_b

plotR1(dose_b, inhibition_b)

## Loewe additive effect estimation

f <- function(E, doseA, doseB){

# Loewe additivity equation

# 1 = (da / Dxa) + (db / Dxb) = (da / f^-1(fa(x))) + (db/f^-1(fb(x)))

# x is equieffective dose

# doseA is actual dose of drug A

# doseB is actual dose of drug B

mina <- estimates_a[[1]]

da <- estimates_a[[2]]

pa <- estimates_a[[3]]

minb <- estimates_b[[1]]

db <- estimates_b[[2]]

pb <- estimates_b[[3]]

doseA / R1inv(E, mina, da, pa) + doseB / R1inv(E, minb, db, pb) - 1

}

dx <- data.frame(A = rep(dose_a, length(dose_b)), B = as.vector(matrix(rep(rev(dose_b), length(dose_a)), byrow = TRUE, nrow = length(dose_a))))

dx <- as.matrix(dx)

Loewe_values_NA <- apply(dx, 1, function(r)

{

tryCatch(uniroot(function(x) f(x, r["A"], r["B"]), c(0.00001, 99.99999))$root, error = function(e) return(NA))

}

)

Loewe_values_NA

Loewe_matrix_NA <- matrix(Loewe_values_NA, nrow = length(dose_b), ncol = length(dose_a), byrow = TRUE, dimnames = list(rev(dose_b), dose_a))

Loewe_matrix_NA["0", "0"] <- 100

Loewe_matrix_NA

Loewe_values <- apply(dx, 1, function(r)

{

tryCatch(uniroot(function(x) f(x, r["A"], r["B"]), c(0.00001, 99.99999))$root, error = function(e) return(100))

}

)

Loewe_values

Loewe_matrix <- matrix(Loewe_values, nrow = length(dose_b), ncol = length(dose_a), byrow = TRUE, dimnames = list(rev(dose_b), dose_a))

Loewe_matrix["0", "0"] <- 100

Loewe_matrix

difference_matrix <- Loewe_matrix - data_matrix

dimnames(difference_matrix) <- list(rev(dose_b), dose_a)

difference_matrix

## calculation of combination index according to Lehar's method

# dilution factor of drug a

dfa <- dose_a[length(dose_a)] / dose_a[length(dose_a) - 1]

dfa

# dilution factor of drug b

dfb <- dose_b[length(dose_b)] / dose_b[length(dose_b) - 1]

dfb

CI <- (log(dfa) * log(dfb)* sum(difference_matrix, na.rm = TRUE)) / 100

CI

## graphics

library(gplots)

pdf(paste(name, ".pdf", sep = ""))

# experimental value matrix

f1 <- heatmap.2(data_matrix, dendrogram ="none", col=colorRampPalette(c("dodgerblue1", "navy"))(10), Rowv = NA, Colv = NA, cellnote = data_matrix, key = TRUE, density.info = "none", trace = "none", notecol = "white", labRow = rev(dose_b), labCol = c(dose_a), xlab = name_a, ylab = name_b, cexRow = 1.2, cexCol = 1.2)

# expected value matrices

f2a <- heatmap.2(Loewe_matrix_NA, dendrogram = "none", col=colorRampPalette(c("dodgerblue1", "navy"))(10), Rowv = NA, Colv = NA, cellnote=round(Loewe_matrix), key= TRUE, density.info = "none", trace = "none", notecol = "white", labRow = rev(dose_b), labCol = c(dose_a), xlab = name_a, ylab = name_b, cexRow = 1.2, cexCol = 1.2)

f2b<- heatmap.2(Loewe_matrix, dendrogram = "none",col = colorRampPalette(c("dodgerblue1", "navy"))(10), Rowv = NA, Colv = NA, cellnote = round(Loewe_matrix), key = TRUE, density.info = "none", trace = "none", notecol = "white", labRow = rev(dose_b), labCol = c(dose_a), xlab = name_a, ylab = name_b, cexRow = 1.2, cexCol = 1.2)

# interaction matrices

f3 <- heatmap.2(difference_matrix, dendrogram = "none", col = greenred(10), Rowv = NA, Colv = NA, cellnote = round(difference_matrix), key = TRUE, density.info = "none", trace = "none", notecol = "white", labRow = rev(dose_b), labCol = c(dose_a), xlab = name_a, ylab = name_b, cexRow = 1.2, cexCol = 1.2, symbreaks = T)

f4 <- heatmap.2(difference_matrix, dendrogram = "none", col = greenred, Rowv = NA, Colv = NA, cellnote = round(difference_matrix), key = TRUE, density.info = "none", trace = "none", notecol = "white", labRow = rev(dose_b), labCol = c(dose_a), xlab = name_a, ylab = name_b, cexRow = 1.2, cexCol = 1.2, symbreaks = T, breaks = c(seq(-25, 25, 5)))

dev.off()
